# Supplementary material for: Comparison of Methods for Incorporating Related Data When Developing Clinical Prediction Models: A Simulation Study
Source: Biom J. 2026 Aug 27;68(5):e70166. doi: 10.1002/bimj.70166 (PMC13520826; doi:10.1002/bimj.70166)
Supplement: Supplementary file 2 [file BIMJ-68-e70166-s001.zip › third submission code review - R2/CASESTUDY_REALDATA_RESULTS/results/FIGURE8(a)-Bootstrap Apparent Metrics.pdf]

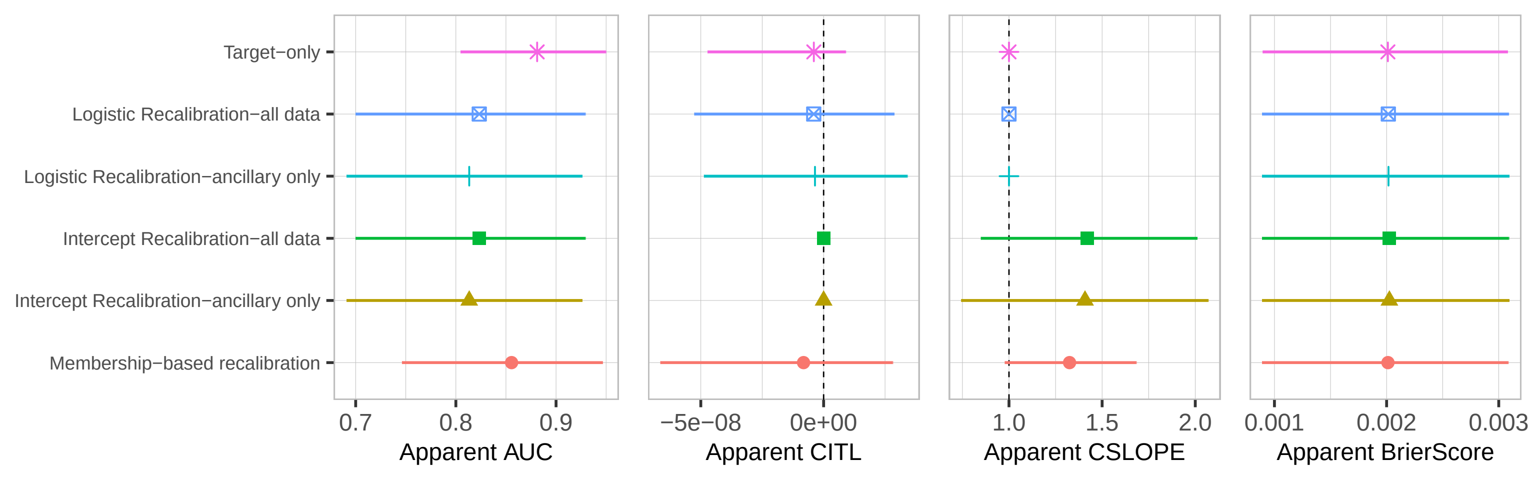

Target−only
Logistic Recalibration−all data
Logistic Recalibration−ancillary only
Intercept Recalibration−all data
Intercept Recalibration−ancillary only
Membership−based recalibration
0.7
0.8
0.9
Apparent AUC
−5e−08
0e+00
Apparent CITL
1.0
1.5
2.0
Apparent CSLOPE
0.001
0.002
0.003
Apparent BrierScore
